# Mitochondrial complex III activity: from invasive muscle biopsies to patient-friendly buccal swab analysis

**DOI:** 10.1038/s41598-023-36741-w

**Published:** 2023-06-14

**Authors:** Tim Somers, Neeltje A. E. Allard, Sailay Siddiqi, Margit C. M. Janssen, Maria T. E. Hopman, Wim J. Morshuis, Frans G. M. Russel, Silvie Timmers, Tom J. J. Schirris

**Affiliations:** 1 Department of Cardiothoracic Surgery, Radboud University Medical Center, Geert Grooteplein Zuid 10, P.O. Box 9101, 6500 HB Nijmegen, The Netherlands; 2 Department of Pharmacology and Toxicology, Radboud University Medical Center, Radboud Institute for Molecular Life Sciences, Nijmegen, The Netherlands; 3 Radboud Center for Mitochondrial Medicine, Radboud University Medical Center, Radboud Institute for Molecular Life Sciences, Nijmegen, The Netherlands; 4 Department of Physiology, Radboud Institute for Health Sciences, Radboud University Medical Center, Nijmegen, The Netherlands; 5 Department of Human and Animal Physiology, Wageningen University, Wageningen, The Netherlands

**Keywords:** Diagnostic markers, Diagnostic markers, Laboratory techniques and procedures

## Abstract

Drug-induced mitochondrial dysfunction is a common adverse effect, particularly in case of statins—the most prescribed drugs worldwide. These drugs have been shown to inhibit complex III (CIII) of the mitochondrial oxidative phosphorylation process, which is related to muscle pain. As muscle pain is the most common complaint of statin users, it is crucial to distinguish it from other causes of myalgia to prevent unnecessary cessation of drug therapy. However, diagnosing CIII inhibition currently requires muscle biopsies, which are invasive and not practical for routine testing. Less invasive alternatives for measurement of mitochondrial complex activities are only available yet for complex I and IV. Here, we describe a non-invasive spectrophotometric method to determine CIII catalytic activities using buccal swabs, which we validated in a cohort of statin and non-statin users. Our data indicate that CIII can be reliably measured in buccal swabs, as evidenced by reproducible results above the detection limit. Further validation on a large-scale clinical setting is recommended.

## Introduction

Mitochondrial myopathy is typically characterized by morphologically and biochemically impaired mitochondria, which results in decreased energy production. These aberrations mostly have a genetic origin, however, many drugs can also induce mitochondrial dysfunction, of which statins are a clear example^1^.

Statins are world’s most prescribed drugs for the treatment of cardiovascular disease with over 180 million users worldwide, but their use is also associated with various side-effects^2,3^. Muscle complaints are reported in 7–29% of all users, varying from common muscle stiffness up to rare life-threatening cases of rhabdomyolysis^2,4–6^. We have previously shown that statins inhibit mitochondrial complex III (CIII)^7^ and CIII activity is inversely correlated with muscle pain intensity in symptomatic statin users^8^. Hence, these findings suggest CIII activity may be useful for identifying statin-induced muscle symptoms (SAMS), as currently no objective or definitive diagnostic test exists^9–11^. There are many different definitions for SAMS and variable protocols available that test how likely muscle complaints are caused by statins. These rely on subjective questionnaires (e.g. SAMS-CI) or (indirect) laboratory abnormalities (e.g. significant elevated CK or liver enzymes)^10–15^. A more specific test to identify SAMS would be of special interest in the context of the recently published meta-analysis suggesting that most reported muscle complaints are not due to statin use^16^. Other factors like comorbidities (e.g. hypothyroidism, rheumatic polymyalgia, vitamin D deficiency), recent intense physical activity or trauma, viral infection, and polypharmacy have been shown to induce muscle pain and should be distinguished from the pain caused by statin use^12,15,17–19^. This distinction is especially important, because nonadherence to statin therapy puts a high burden on cardiovascular morbidity and mortality^11^.

CIII catalytic activity, like other complexes of the oxidative phosphorylation system (OXPHOS)^20,21^, is typically determined by means of colorimetric measurements in muscle biopsies or cellular fractions^22^. Muscle biopsies cause significant subject burden^23^ and a less invasive alternative for measurement of complex I and IV activity is available by performing spectrophotometric and immunocapture assays in cells recovered from buccal swabs^24–27^. The latter method seems to show a good correlation with more traditional methods, with R^2^ ranging from 0.49 to 0.99 for activity measurements of complex I and IV^24–27^. However, a similar assay for CIII is missing. Here, we adapted and validated a method to measure CIII activity based on enzymatic cytochrome C reduction^28^, such that it can be used in spectrophotometer microplate readers using non-invasive buccal swab samples. The sensitivity and validity of this measurement was tested in a large group of control subjects and statin users with and without muscle complaints.

## Results

### Spectrophotometric measurement of CIII activity in a 96-well plate is not accurate enough

**Figure 1:**
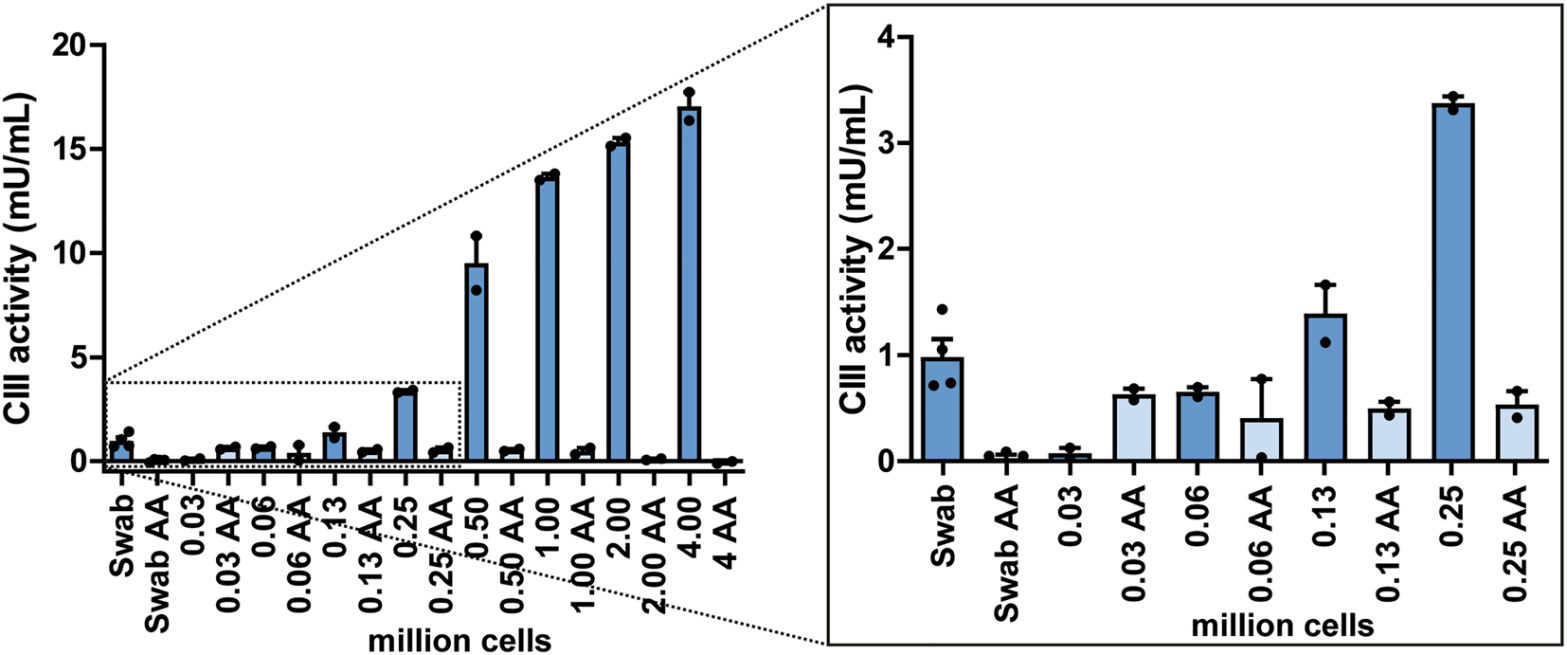
CIII activity in dilution series of HeLa cells compared to buccal swab samples from a non-CIII deficient person based on lack of symptoms. The CIII activity expressed in mU mL^−1^; mean ± SEM with individual data points plotted. The dilution series for HeLa cells are from one vial, the number of cells (in millions) depicted on the x-axis. One sample of four swabs was used as mentioned before. AA (antimycin A, light blue) was the inhibited or basal cytochrome C reduction capacity for each condition. If the concentration-dependent curve reached plateau, measurements were considered equal to background (= around 62,500 cells).

In the first set-up, CIII activity was measured in buccal swabs from persons without a CIII deficiency, by means of an enzymatic cytochrome C reduction assay, measured spectrophotometrically in a 96-well plate. This assay was based on the preliminary data of supplementary Fig. 1. By adding the CIII inhibitor antimycin A (AA), the cytochrome C reduction independent of this complex (background) was measured. Because of the small sample volume relative to the larger wells, this resulted in CIII activities with a high level of inter- and intra-experimental variability (results not shown). Therefore, HeLa cells were cultured to create dilution series with mitochondria in a more controlled fashion, which were kept more constant between the different experiments. The HeLa dilution series showed a gradual decline in CIII activity with decreasing cell number, whilst the background (basal cytochrome C reduction) remained the same (Fig. 1), and with little variation within the samples. With a five times dilution (approximately 0.25 million cells) the curve flattened (see enlarged part of Fig. 1) and reached the detection limit of the assay. The CIII activity measured in the buccal swab was similar to seven times diluted HeLa cells (approximately 62,500 cells/sample), and thus fell outside the detection limit.

### Increased yield of mitochondrial fraction improves accuracy of fluorescent CIII activity measurement

**Figure 2:**
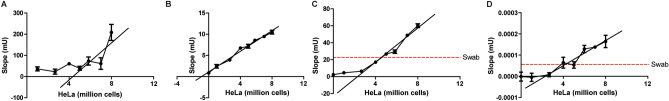
Fluorescent and spectrophotometric based CIII activities in HeLa dilution series. Fluorescence measurement in 96-well plate (**A**) and 384-well plate (**B**,**C**) and spectrometric measurements in 384-well plate (**D**) of HeLa cell dilution series (in millions of cells) and swab sample of four pooled swabs. Results are presented as mean ± SEM. Black lines are the linear regressions curves drawn through all points. Dilutions (in millions of HeLa cells) below the intersection of this regression line and the x-axis represent the minimal working range. The red dotted line is the level of CIII activity for a swab sample of a non-CIII deficient person. If this line intersects with the regression line above the intersection with the x-axis, the results were considered to fall above the detection limit of the assay.

The dilution series of HeLa cells showed that the CIII spectrometric enzyme activity assay becomes inaccurate around the plateau phase of 62,500 cells/sample. As the CIII activity in buccal swabs was around this level and thus unreliable, fluorescence measurements were adopted to improve accuracy at lower detection limits. Figure 2A depicts the results of CIII activity in a dilution series of HeLa cells measuring the reduction of cytochrome C with fluorescence. The determined slope crosses the x-axis around a 4–5 million HeLa cells, suggesting anything below this level is inaccurate. The swabs from persons without a CIII deficiency (not depicted) had a CIII activity comparable to 0.5–1 million HeLa cells. To further improve the detection limit, due to the limited cell content in the buccal swabs, the ratio between reaction mixture and mitochondrial content was raised. To avoid having too little volume in each well (< 100 µL), the well size was also reduced. Increasing the cellular and mitochondrial fraction (from 1:2 to 1:1) by use of a 384-well plate resulted in a linear relationship between CIII activity and the HeLa cell dilution series (Fig. 2B). A similar response was observed for CIII activities in biologically independent replicates (Fig. 2C).

### Increased yield of mitochondrial fraction in a 384-well plate gives reliable spectrophotometric measurement of CIII activity

**Figure 3:**
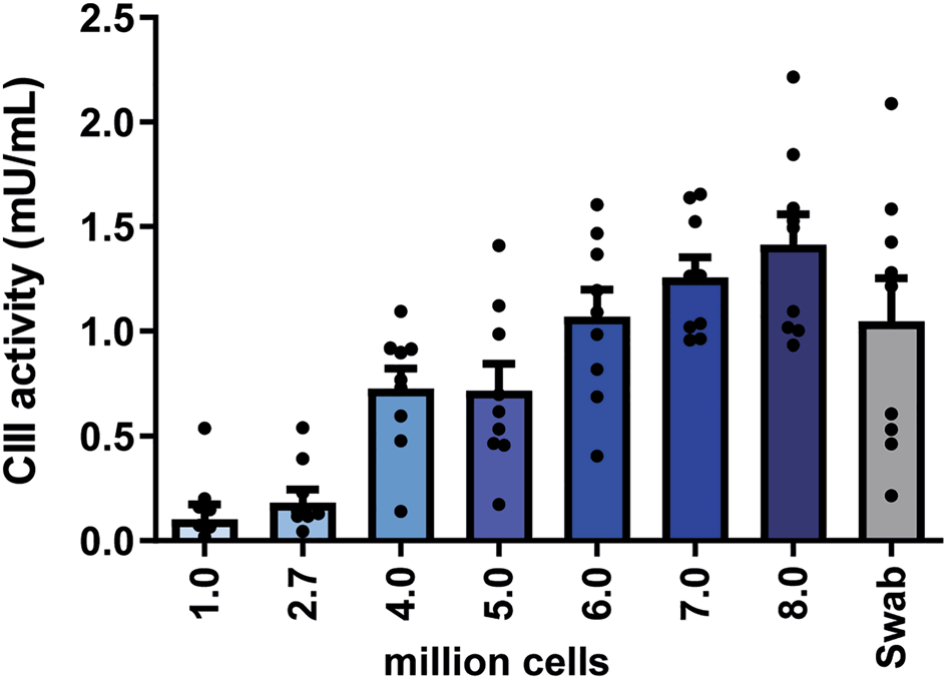
CIII activities in HeLa dilution series in 384-well plate measured with spectrophotometry. Spectrophotometric measurements of HeLa cell dilution (in millions of cells) and four swabs pooled as swab sample in a 384-well plate. Pooled results of three biologically independent experiments. Results are presented as mean ± SEM with individual data points plotted. The swab sample falls within the working range of the assay if the bar is above the lowest values of HeLa cells that showed no further decline in CIII activity after increased dilutions (background from dilution 0.333 onwards).

Since the CIII inhibitor AA, which is necessary to block the enzymatic cytochrome C reduction, revealed autofluorescent properties (data not shown), we switched back to the spectrophotometric CIII assay. To increase the mitochondrial concentration a 384-well instead of 96-well plate was chosen (Fig. 2D). Results from three biologically independent experiments showed a concentration-dependent CIII activity comparable to the fluorescent assay (Fig. 3). The results obtained with the buccal swab samples from persons with non-deficient CIII were also clearly above the detection limit in all three experiments (CIII activity above plateau phase in HeLa cells).

**Figure 4:**
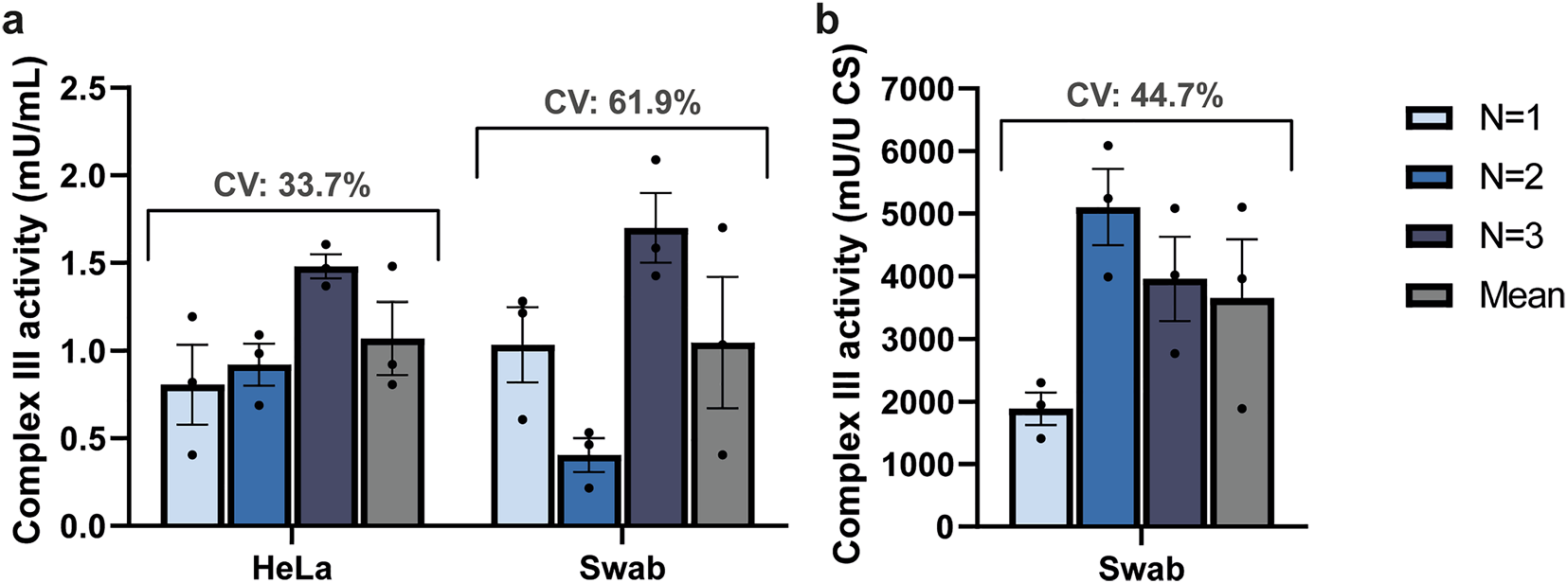
Coefficient of variation for spectrophotometric CIII activities in HeLa cells and swabs. Spectrophotometric measurements for CIII activity of HeLa cells and swab samples (**a** uncorrected) and corrected for citrate synthase activity in swab samples (**b**). Results are from three individual experiments (N = 1–3) and pooled (mean). Results are presented as mean ± SEM with individual data points plotted. Inter-assay coefficient of variation (CV) is shown. *CS* citrate synthase.

The inter- and intra-assay coefficient of variation (CV) for the HeLa cells was 33.7% and 26.5%, respectively. The intra-assay CV for swabs was in line with HeLa cells (32.5%). However, the inter-assay CV for buccal swabs was rather high (61.9%) (Fig. 4). This variability was corrected by citrate synthase (CS) measurements, a well-established method to adjust for the variability in cell number and mitochondrial content obtained from buccal swabs^23,24,29^. After CS correction the inter-assay CV for buccal swabs reduced to 44.7%. Correction by protein levels using the Bradford assay or the Pierce™ BCA protein kit (Thermofisher) were inferior to CS and therefore not further applied (results not shown)^30,31^.

### CIII activity assay validation in statin users

CIII activity was examined in 69 participants that received statin treatment, with (n = 35) and without muscle complaints (n = 34) and non-statin control subjects (n = 31). Thirteen participants were excluded because of an unmeasurably low CIII activity, including nine statin users (three symptomatic and six asymptomatic) and four controls. Baseline characteristics of the remaining 87 participants are shown in Table 1. The symptomatic statin users were significantly younger compared to both control and asymptomatic statin users (p = 0.022). All other baseline characteristics were similar between groups.

**Table 1:** Characteristics of the study participants.

	Non-statin users (control)	Asymptomatic statin users	Symptomatic statin users	*P *value
Number (N)	27	28	32	
Sex				0.422/1.000
Female	9	6	6	
Male	18	22	26	
Age (years)	66.4 ± 1.1	66.4 ± 1.3	62.3 ± 1.3	0.022/0.028
BMI (kg m^−2^)	26.4 ± 0.7	26.8 ± 0.6	26.7 ± 0.6	0.920/0.951
MAP (mmHg)	99.0 ± 2.0	99.3 ± 2.0	101.5 ± 1.6	0.572/0.389
Total MET (squash)	5190 ± 540	5520 ± 610	5570 ± 720	0.905/0.960
Walking distance (km)				0.224/0.135
30	18	18	15	
40	7	10	13	
50	2	0	4	
Statin				0.494
Simvastatin		15	12	
Atorvastatin		6	11	
Rosuvastatin		4	7	
Pravastatin		2	2	
Fluvastatin		1	0	
Duration statin use (months)	–	96.2 ± 10.5	86.5 ± 11.6	0.544

Data is indicated as N or mean ± SEM. P value for control vs. statin users and asymptomatic vs. symptomatic. *BMI* body mass index, *MAP* mean arterial pressure, *MET* metabolic equivalent of task, *ns* non-significant.

**Figure 5:**
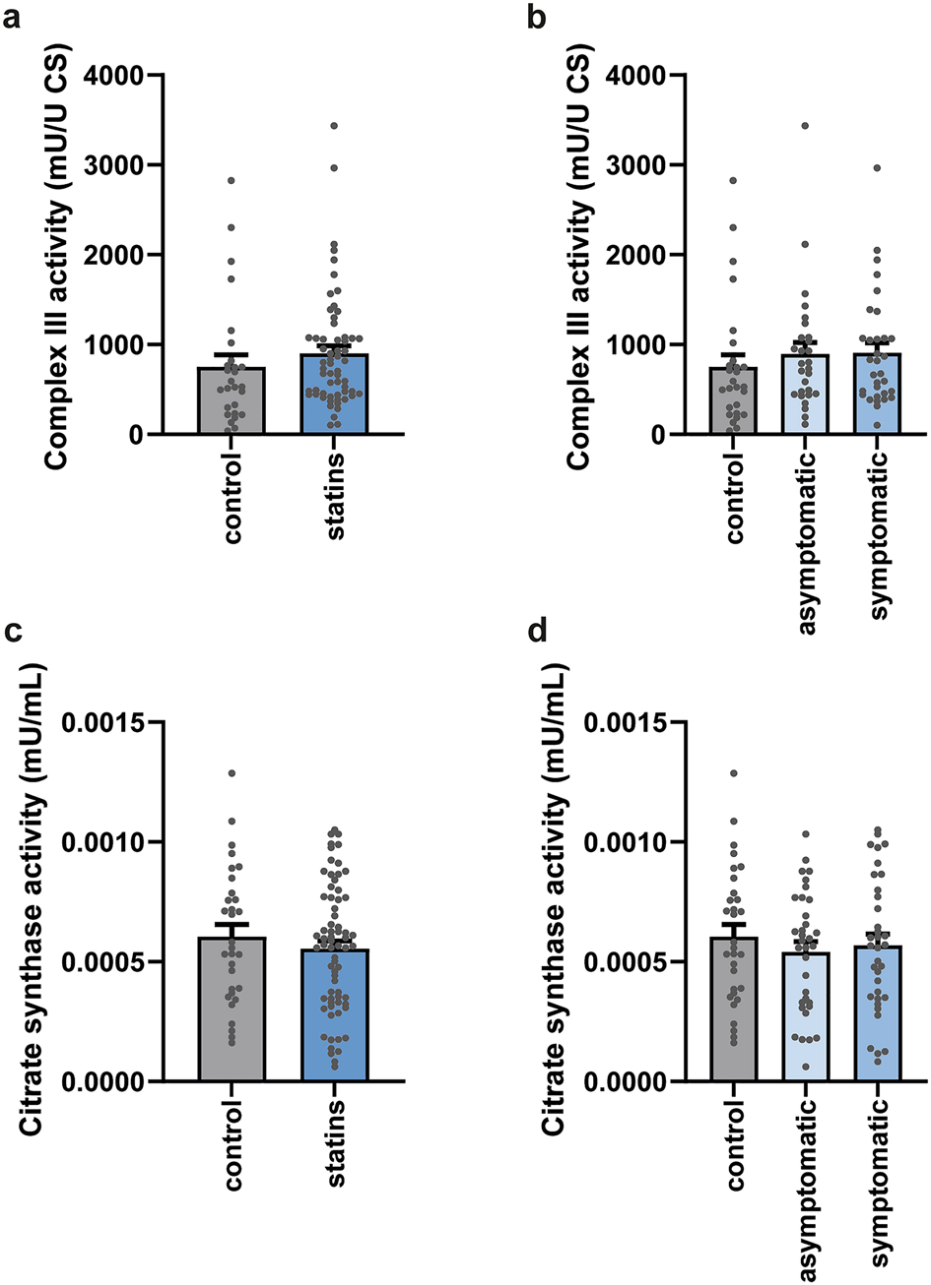
CS activity corrected CIII activity and CS activity in buccal swab samples from participants of the Nijmegen Four Days Marches. (**a**) CIII and (**c**) CS in patients treated with statins (n = 60) and controls (n = 27) and (**b**) CIII and (**d**) CS in statin users with muscle complaints (n = 32), without complaints (n = 28), and non-statin users (n = 27). Activity was measured as the absorbance from the cytochrome c reduction reaction and normalised by citrate synthase (CS) activity or as CS activity alone. No statistical differences were found. Data is presented as mean ± SEM with individual data points plotted.

Mean CIII activities corrected for CS were not different between statin users and control subjects (respectively 900 ± 80 versus 750 ± 130 mU U^−1^ CS; p = 0.326). There was also no difference in CS corrected CIII activity between symptomatic and asymptomatic statin users (respectively 910 ± 110 and 890 ± 130 mU U^−1^ CS; p = 0.930), and no difference between either statin group and the control group was observed (respectively p = 0.362 and p = 0.442) (Fig. 5a,b). CS activity did not differ significantly between control and statin users (both symptomatic as asymptomatic) (Fig. 5c,d). However, CIII activity did correlate significantly with CS activity (p < 0.001, r = 0.565).

## Discussion

Statin-induced muscle complaints are an important reason for statin discontinuation and appear to be associated with mitochondrial dysfunction, which could be due to statin-induced CIII inhibition^2,7,32^. Current evaluation of mitochondrial complex deficiencies or inhibition requires invasive muscle biopsies^33,34^. New non-invasive approaches to investigate complex-specific mitochondrial dysfunction are being explored^24,26^. Buccal swabs have been used to measure changes in mitochondrial complex I and IV activities after drug treatment. Therefore, we developed a test protocol for a mitochondrial CIII function assay using buccal swabs, which was validated in a population with and without statin-induced muscle complaints.

The CIII activity measured using buccal swabs from persons with non-deficient CIII based on symptomatology produced consistent outcomes above the detection limits of the assay (> 0.2 mU mL^−1^). This detection limit was based upon CIII measurements from dilutions of controlled HeLa cell numbers. It was set just above the lowest CIII values that showed no further decline in CIII activity (representing background measurement). Correction for CS activity further reduced the assay variability in buccal swabs. The samples from statin and non-statin users, used for the validation experiment, yielded a remarkable but non-significantly higher CIII activity in statin users compared to non-users. This was opposite to what was seen in muscle biopsies of statin users^7,8^ and was unrelated to mitochondrial content, as no difference was seen in CS activity between statin users and non-users^29^. Also, there was no correlation between CIII or CS activity and statin use or muscle complaints.

The CIII spectrophotometric enzyme activity assay was based upon previously reported methods developed for other mitochondrial complexes (I and IV)^24,26,27^. For CI and CIV these include dipstick assays using immunocapturing of CI and CIV that are subsequently used to drive an enzymatic reaction of which the coloured precipitate is quantified^26^. A similar dipstick method is lacking for CIII, which is why we developed another assay to measure CIII activity. We based the extraction buffer solution and protocol for preparation of mitochondrial fraction on these dipstick assays, with the addition of a pottering step to further increase the yield (data not shown) and consequently improve sensitivity^35,36^. A disadvantage of our assay is that it is not immunocapture based, which could explain the higher coefficient of variations we observed (around 39% vs. 10% in literature) and the overlapping CIII activity between statin users and non-users^26,37,38^.

A recent review article on the sample selection and choice of test to study mitochondrial bioenergetics in human material^34^, also stresses the limitations of the sample size when choosing for an alternative to muscle biopsy. They address the challenge of many promising techniques using platelets or leukocytes due to failure to increase reproducibility or difficulty to scale up. Two improvements to the buccal swab assay could be respirometry in frozen samples and removal of bacteria from the samples, as bacterial contamination has been shown to disturb measurements and decrease specificity^34,39–41^.

Limitations of this study are that we could not obtain muscle biopsies from statin users and non-statin using controls for cross-tissue validation of CIII activity. Careful interpretation of the data is also necessary due to the high turn-over rate of the oral epithelial layer. Statin-induced CIII inhibition is partly a consequence of long-time exposure of muscle tissue to statins and hence associated with intramyocellular statin accumulation due to slower protein turnover in this tissue^42,43^. On the other hand, the oral epithelial layer with buccal mucosa cells has a high cellular turn-over rate of approximately 2.7 h^44^. As a consequence, limited time is available for statins to accumulate, and it thus remains questionable whether buccal swabs are an adequate surrogate for muscle biopsies in the diagnosis of statin-induced CIII inhibition.

Buccal swab sampling and processing could also be optimized further. The presence of traces of red blood cells in the samples was associated with higher CIII and CS activity levels. Since the absorbance for CIII was measured at the wavelength that matched both cytochrome c reduction as well as red blood cells’ haemoglobin (550 nm, red light), the presences of blood could have falsely increased the signal^45^. As CS measurements uses a wavelength that also is in close proximity with the absorbance of haemoglobin (412 vs 550 nm), it is insufficient to correct for traces of blood^45^. Consequently, samples containing blood traces (haemoglobin) had to be excluded on visual inspection. Another factor is the processing phase of the buccal swabs up to mitochondrial isolation, as this has been adapted over time with addition of pottering and longer spinning times to improve the assay sensitivity. The validation samples were however processed according to one of the first protocols and therefore their yield might have been too low resulting in measurements below the assay detection limits. Furthermore, a total of 13 patients (13%) were excluded due to immeasurably low CIII activities, though they were evenly distributed among statin users (three symptomatic and six asymptomatic) and controls (four).

This study is a step toward the future development of a more sensitive dipstick assay. The current assay uses the less invasive buccal swabs to measure mitochondrial CIII activities consistently. Including an immunocapture method, together with improving sample acquisition without blood and purification of samples with pottering, could potentially further decrease the assay variation. The enzymatic reaction and spectrophotometry measurements acquired here are also applicable in a dipstick assay as are applied for complex I and IV^24,26^. Additionally, it is worthwhile to look into the possibility of measuring in frozen samples and to optimize bacterial removal. It might then be recommended to use known CIII-deficient patients to evaluate the correlation with this previous diagnosed mitochondrial defect as was done for CI and CIV^24,26^ or assess CIII activity by swabs from patients with biopsy-proven drug-induced CIII inhibition in skeletal muscle. It is debated whether statins are the cause for the reported SAMS^16,46^. Optimalisation of the current assay could help to distinguish between muscle symptoms due to statin-induced CIII inhibition or to other causes^7^.

## Conclusion

The currently described protocol can adequately measure levels of mitochondrial complex III activity in a non-invasive manner, although it needs further optimization and validation in a clinical setting.

## Methods

### Reagents

2-Amino-2-(hydroxymethyl)propane-1,3-diol (TRIS UltraPure) (Invitrogen, #15504020); Antimycin A (Sigma-Aldrich, #A8674); Cytochrome C (Sigma-Aldrich, #C7752); 2,3-dimethoxy-5-methyl-6-decyl-1,4-benzoquinon (Decylubiquinone) (Enzo Life Sciences, #BML-CM115-0010); Potassium phosphate dibasic (Sigma-Aldrich, #P3786-M); Potassium phosphate monobasic (Acros Organics, #205925000); Dimethyl sulfoxide (DMSO) (Sigma-Aldrich, #276855); Ethylenediaminetetraacetic acid, 2Na (Na-EDTA) (Merck, #324503); Heparin (Sigma-Aldrich, #H3393); Ethanol (Merck, #100983); Potassium borohydride (Sigma-Aldrich, #60080); Sodium azide (Sigma-Aldrich, #S2002); Sucrose (Acros Organics, #220900025); Tween-20 (Sigma-Aldrich, #P1379); Hydrochloric acid fuming (37%) (Merck, #100317); Potassium hydroxide (Merck, #105032); n-dodecyl β-d-maltoside (Sigma-Aldrich, #D4641); Sodium chloride (Sigma-Aldrich, #S9888); HEPES (Sigma-Aldrich, #H3375); cOmplete™, mini, EDTA-free Protease inhibitor cocktail (Sigma-Aldrich, #11836170001); DNA/RNA Buccal Swabs (Isohelix™, #SK-1S); DMEM, high glucose, GlutaMAX™ Supplement, pyruvate (Gibco™ Life Technologies, #31966047); Fetal bovine serum (FBS; Greiner Bio-One, Alpen aan de Rijn, Netherlands, #758093); Phosphate buffered saline 1× (PBS; Laboratory support and medium preparation LEM, RIMLS, Radboudumc, Nijmegen, The Netherlands); 0.05% Trypsin–EDTA (1×) (Gibco™ Life Technologies, #25300054).

### Cell culture

HeLa cells (American Type Culture Collection, CCL-2) were maintained at 37 °C in a humidified atmosphere of 5% (v/v) CO2 in Dulbecco’s Modified Eagle’s medium (DMEM) containing 25 mM glucose, GlutaMAX™, 1 mM pyruvate supplemented with 10% (v/v) FBS. If confluent, cells were washed with PBS, dissociated with Trypsin and after 6–7 min incubation at 37 °C passaged in a ratio of 1:10. Cells were also spun down (5 min, 1000 g) and resuspended in DMEM for counting. Cells were divided in Eppendorf 1.5 mL tubes, approximately 8 million cells/tube and spun down again prior to snap freezing in liquid nitrogen. These cells were stored at − 20 °C for experiments.

### Buccal swab sample preparation

For every patient at least 4 buccal swabs are obtained. It is important the mouth is rinsed before the samples are collected to prevent any food or drink residues disturbing the sample. Next each swab is put in 175 µL extraction buffer (pH 7.4) consisting of lauryl maltoside (dodecyl maltoside) (1.5% w/v), 100 mM NaCl, 25 mM HEPES and one protease inhibitor cocktail tablet. Each buccal swab is briefly vortexed (approximately 20 s) and spun down in a 4 °C cooled centrifuge for 5 min, 8500*g*. The supernatants and pellets of four buccal swabs are combined and suspended in 1 mL of 10 mM Tris–HCl (pH 7.4). This Tris–HCl-Swab suspension is pottered 6–8 times and then incubated on ice for 20 min. Next the tubes are spun down in a 4 °C cooled centrifuge for 20 min, 16,000*g*. All cellular fractions are collected in the pellet and discarded and the supernatant with mitochondria is used for the following analyses.

### CIII activity measurement

The CIII activity measurements are determined by a spectrophotometric assay based on the following enzymatic reaction: DUH_2_ + cytochrome c (oxidized) → DU + cytochrome c (reduced) + 2H^+^.

For the 96-well configuration: in a 96-well clear F-bottom microplate (Greiner Bio-One, Essen, Germany) 72.5 µL of reaction mixture is pipetted in every well. The reaction mixture is made fresh on the day of experiments and consists of Milli-Q (14.6% v/v), 50 mM potassium phosphate buffer (pH 7.8; 66.4% v/v), 0.3 M NaN_3_ (1.3% v/v), 0.1 M Na-EDTA (1.3% v/v), 2% Tween-20 (2.7% v/v), 1 mM cytochrome C (13.3% v/v) and reduced DUH_2_ (0.4% v/v). Add in the DUH_2_ 5 min prior to usage and incubate on ice to prevent precipitation. 2.5 µL Milli-Q (MQ) or antimycin A (AA; minimal concentration of 10 nM) is added respectively to the activity or blank wells. AA is an inhibitor of CIII and used to measure basal cytochrome C reduction (background) to determine actual CIII activity.

For the 384-well configuration: in a 384-well black/clear bottom microplate (Greiner Bio-One, Essen, Germany) 40 µL of reaction mixture is put in every well. This reaction mixture is without cytochrome C: Milli-Q (16.8% v/v), 50 mM potassium phosphate buffer (pH 7.8; 76.6% v/v), 0.3 M NaN_3_ (1.5% v/v), 0.1 M Na-EDTA (1.5% v/v), 2% Tween-20 (3.1% v/v) and reduced DUH_2_ (0.4% v/v). 5 µL MQ or AA (minimal concentration of 10 nM) is added respectively to the activity or blank wells.

For both assays (after addition of AA or MQ): the plate is put in a Bio-Rad Benchmark Plus plate reader with Microplate Manager 5.2 Build 103 software (Bio-Rad Laboratories B.V., Veenendaal, The Netherlands) to warm-up and mix (25 °C). After 5 min 25 µL (96-well configuration) or 40 µL (384-well configuration) of sample is added. The catalytic reaction was only just started in the 384-well configuration by the addition of 7.7 μL 1 mM oxidized cytochrome c and the formation of reduced cytochrome c was measured at 550 nm at 25 °C in the plate reader. Cytochrome C was already added in the 96-well configuration as component of the reaction mixture. Kinetic measurements were taken every 20 s for a total duration of 20 min.

Experiments were performed in triplicate, the difference obtained between the sample and blank represents the activity of CIII. For this the measurements are plotted on a time curve and a slope is calculated for minimally the first 10 datapoints in Graphpad Prism. Outliers were excluded. The slope is used in the following formula to calculate the CIII activity.

$$\frac{\Delta E/\mathrm{min}}{0.191}\times Dilution\times \frac{T}{x}=\mathrm{mU}/\mathrm{mL} \,complex \,III \,activity$$

ΔE min^−1^: average increase of absorption at 550 nm per minute in the reaction sample; Dilution: dilution of the mitochondrial fraction, if not diluted keep it 1; T: total reaction volume (in µL), usually 92.7 µL; x: volume of added mitochondrial fraction in µL, usually 40 µL; 0.0191 µmol^−1^ cm^−1^: e550nm of reduced cytochrome c.

The average of negative control wells (without cells) is subtracted from all other activity values. Next the absolute activity = activity of experimental wells (MQ) − activity of blank wells (AA). CIII activities are deemed invalid when equal to or below 0.

### Citrate synthase measurements

Citrate synthase (CS) is a unique mitochondrial enzyme, and therefore a marker of mitochondrial mass. It is used as correction factor for the variability in cell content retrieved by buccal swabs. Previous protein measurements were not able to sufficiently lower the coefficient of variation.

CS activity was determined by a spectrophotometric assay, based on the following enzymatic reactions: acetyl-CoA + Oxaloacetate + H_2_O ↔ Citrate + CoASH + H^+^ and CoASH + DTNB ↔ DTNB-SH + CoA.

The supernatant with mitochondrial fraction obtained previously (for CIII measurements) is used and 50 µL added in every well of a 384-well microplate. All reagents are dissolved in 10 mM Tris–HCl (pH 7.6). The assay mixture, containing 2 mL of Milli-Q, 500 µL of 1 mM DTNB and 10.42 µL 10% Triton X-100, is added in 41.7 µL well^−1^. Finally, 5.6 µL of 3 mM acetyl CoA is added. Basal DTNB-SH production was measured at 412 nm for 20 min (kinetic) in a Bio-Rad Benchmark Plus plate reader with Microplate Manager 5.2 Build 103 software. Subsequently, 2.8 μL 10 mM oxaloacetate was added to start the catalytic reaction, and after mixing, DTNB-SH production was measured at 412 nm for another 20 min (kinetic). The difference between the slopes determined for both plotted measurements in GraphPad Prism resulted in the CS activity per sample.

The obtained CIII values are divided by the CS activity values which results in the corrected CIII activity.

### Fluorescence measurements

The previous samples were also measured in the SpectraMax iD5 microplate reader (Molecular Devices; San Jose, CA), to determine the fluorescence of cytochrome C reduction. Wavelengths were 350 nm and 450 nm for excitation and emission, respectively^47^.

### Protocol validation in patients

Hundred adult participants of the 102nd edition of the Nijmegen Four Days Marches (Nijmegen, The Netherlands) were included in the study. Statin users were included if they had used statins ≥ 3 months prior to study participation. Statin users were considered symptomatic or asymptomatic based on the presence, localization and onset of muscle cramps, pain, and/or weakness, using the statin myalgia clinical index score^9^. BMI, hip circumference and blood pressure were measured 1 or 2 days before the event (Table 1). The Short Questionnaire to Assess Health-enhancing physical activity (SQUASH) was used to determine physical activity levels^48^. Participants were asked to collect four buccal swabs. Sample collection was performed for 30 s per swab, on an empty stomach and after rinsing the mouth twice. The buccal swab samples were collected as an additional measurement during a larger trial, which is registered within ClinicalTrials.gov (NCT05011643). The study was approved by the medical ethics committee of the Radboud university medical center and participants provided written informed consent. All methods were performed according to relevant Radboud university medical center guidelines and regulations. Buccal swab sample collection and questionnaires were obtained 1 or 2 days prior to the event.

Samples were placed in 4 °C extraction buffer (see above), vortexed for 20 s, and centrifuged for 5 min (8500*g*, 4 °C). The supernatant was isolated and incubated on ice for 15 min. To isolate the mitochondria, the samples were centrifuged for 12 min (16,000*g*, 4 °C). For each participant the samples were pooled, snap-frozen in liquid nitrogen, and stored by − 80 °C until CIII measurements were performed.

### Statistical analysis

All data is analysed using GraphPad Prism version 5.03 (GraphPad Software, San Diego, CA, USA) and IBM SPSS^®^ Statistics (version 25, Armonk, NY, United States). Results are presented as mean with standard error of the mean (± SEM). CIII activity and CS activity is determined as described above. Data was blank-corrected and normalised using these CS activity measurements.

Intra-assay coefficient of variation is calculated based on the coefficient of variation of each condition per biologically independent experiment. Inter-assay coefficient of variation is calculated based on the mean of three replicates per condition of each experiment.

Patients with a negative mean CIII activity were excluded. Information on statin use was blinded from the researchers until statistical analysis was performed. Differences between statin users and control subjects were analysed using an independent samples t-test. Differences between statin users with muscle symptoms, asymptomatic statin users, and non-statin users were determined with χ^2^ test and one-way ANOVA. Bonferroni post hoc tests were performed to correct for multiple comparisons. An “α”-level of 0.05 was used to determine statistical significance. Correlations were examined using a two-tailed non-parametric Spearman’s rho test and were significant at an 0.01 level.

## Supplementary Information

Supplementary Information.

## Data Availability

All data used in this study are available from the corresponding author on reasonable request.
