# Supplementary material for: Mitochondrial complex III activity: from invasive muscle biopsies to patient-friendly buccal swab analysis
Source: Sci Rep. 2023 Jun 14;13:9638. doi: 10.1038/s41598-023-36741-w (PMC10267125; doi:10.1038/s41598-023-36741-w)
Supplement: Supplementary file 1 — Supplementary Information. [file 41598_2023_36741_MOESM1_ESM.docx]

**Preliminary tests comparing buccal swabs with current standards**

Currently, routine diagnostic complex III (CIII) determinations are performed in muscle biopsies. To allow using CIII activity measurements as a clinically applicable point-of-care test the aim was to develop a CIII dipstick assay like current dipstick assays for complex I and IV (Abcam). The importance of CIII measurements has been shown by the correlation between the severity of statin-induced muscle complaints and the extent of statin induced CIII inhibition^8^. The idea has risen to test patients ahead of starting statin therapy to evaluate their risk on developing CIII inhibition and muscle complaints. Prior to developing such an assay, a pilot study measuring cellular CIII activity in buccal swabs was performed as was done before for complex I and IV^26^. This pilot study demonstrated the feasibility to detect CIII activity in salivary samples. Skin fibroblasts and murine myoblasts were cultured, and buccal swabs taken from a healthy control followed by CIII measurements. As is shown in **supplementary figure 1a**, measured CIII activity was lower than measured in muscle tissue, however, importantly higher than in skin tissue. Skin tissue has been shown to be suitable to measure CIII at a detectable level (see **figures S1b+c**). Fibroblasts were cultured and CIII activities were measured for different dilution series. For every dilution, protein levels were quantified. CIII activity reached steady state at protein levels that were seen in salivary samples (**Figure S1b**). Finally, four different human skin fibroblast cell lines were cultured, including two different CIII-deficient cell lines (P6748 and P6147) and two different healthy control cell lines (C6855 and C5120). The CIII assay was sufficiently accurate to measure significant differences between CIII-deficient fibroblasts and controls (**Figure S1c**). The results were also in line with current routine diagnostics (dotted lines in **figure S1c**). However, these measurements required further optimization of sampling technique and correction for variation in mitochondrial mass to reduce inter- and intra-experimental variability.


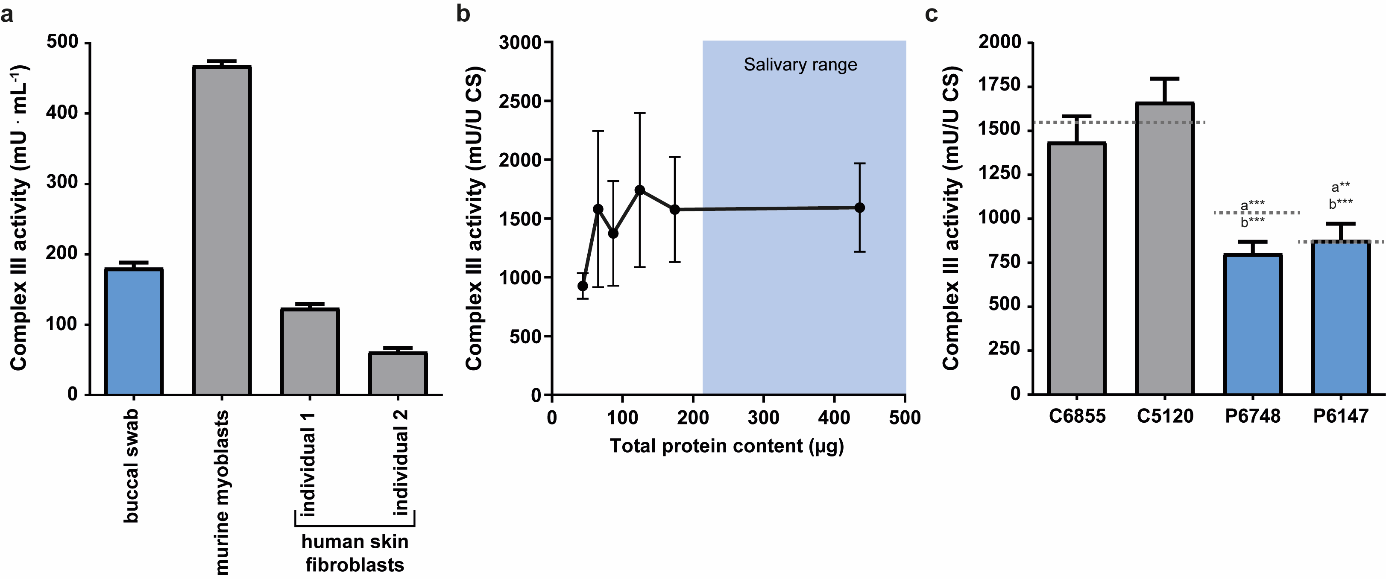


**Figure S1 – Complex III (CIII) activity in buccal swabs and fibroblasts of controls and CIII-deficient cell lines.** (**a**) CIII activity of buccal swabs compared to murine myoblasts and human skin fibroblasts. (**b**) stability of CIII activity per protein content measured in dilution series of fibroblasts, and protein content of saliva is marked with the blue shaded box. (**c**) CIII activity measured with buccal swabs in skin fibroblasts of healthy controls (C6855 and C5120) compared to known CIII-deficient persons (P6748 and P6147). The level of routine diagnostic determination is marked by the dotted line. Data is pooled for three biologically independent experiments and presented as mean±SEM. Statistical differences were determined using one-way ANOVA with Bonferroni post hoc test. **p<0.01 and ***p<0.001.
